# Persons and Genes. Is a Gene-Centered Evolutionary Psychology Compatible with a Person-Oriented Approach to Psychological Science?

**DOI:** 10.1007/s12124-020-09548-x

**Published:** 2020-05-29

**Authors:** Lars-Gunnar Lundh

**Affiliations:** Department of Psychology, Lund University, Box 213, 221 00 Lund, Sweden

**Keywords:** Person-oriented approach, Holism, Interactionism, Idiographic, Gene-centered, Evolutionary psychology

## Abstract

According to Zagaria et al. (2020), evolutionary psychology may be the meta-theory that is needed if psychological science is to enter a paradigmatic stage. Other writers have suggested that what is needed is a person-oriented approach, which focuses on the person as a complex system that needs to be studied (1) as a whole (holism), (2) as an intentional agent in interaction with its environment (interactionism), and (3) in terms of his or her individual characteristics and development (idiographic focus). The purpose of the present paper is to discuss the compatibility of these two suggestions. A brief analysis of some formulations central to Dawkins’ gene-centered approach (e.g., “the intricate interdependence of genes”, and the dependence of genes on their environment) suggests that it is quite compatible with holism and interactionism; and applications such as genetic genealogy illustrate the possibility of a person-oriented genetics. It is argued that these two perspectives are not only compatible, but also complementary. Without a complement in the form of a person-oriented perspective, a gene-centered evolutionary psychology will at best be able to produce a general understanding of the psychological potentials that inhere in the human gene pool. It will not, however, lead to any understanding of the unique profiles of psychological potentials that are produced by a re-combination of autosomal DNA at the origin of each specific individual person, and that develop over time in interaction with the environment. The latter requires that the gene-centered view is complemented with a person-oriented approach.

## Introduction

According to Zagaria et al. (2020), evolutionary psychology may be the meta-theory that is needed if psychological science is to enter a paradigmatic stage. The kind of evolutionary psychology that they have in mind is not evolutionary psychology in a “narrow sense”, defined in terms of specific theories (such as the computational postulation, the massive modularity hypothesis and the notion of EEA), but evolutionary psychology in a “broad sense” as a pluralistic theoretical framework that may potentially serve to integrate a wide variety of theoretical approaches that posit universal and inborn tendencies in human beings (such as homeostasis, drives, individualization, and self-actualization).

The main strength of evolutionary psychology, as they see it, is its capacity to resolve the nature nurture dialectics. In their view, evolutionary psychology is based on two theoretical foundations, one nested in the other. The first foundation is the process of natural selection itself, which chooses which components are part of human innateness. Here they refer to Dawkins’ *gene-centered view of evolution* which implies that the selection process does not act on individual organisms or species, but on genes, defined as “any portion of chromosomal material that potentially lasts for enough generations to serve as a unit of natural selection” (Dawkins 2016, p. 36). The second foundation is the importance of the social environment in shaping our psychological functioning, by means of human beings’ evolutionarily designed capacities for cooperation, communication, mentalization, sharing of attention, etc. (e.g., Tomasello 2019).

Other writers have suggested that what psychological science needs is a person-oriented approach, where the person is treated as an integrated whole that develops and functions as an intentional agent in interaction with the environment (e.g., Bergman and Andersson 2010; Bergman and Lundh 2015; Bergman and Magnusson 1997; Magnusson 1999, 2001). It has been argued (Lundh 2019) that present-day psychological sciences suffers from a crisis that requires a shift to such a person-oriented paradigm.

The purpose of this commentary is to discuss the compatibility of these two suggestions. After a brief discussion of the concept of person and what characterizes a person-oriented approach to psychological science, I will turn to a discussion of how the gene-person relationship may be conceptualized, and to a discussion of the compatibility of a person-oriented approach with evolutionary psychology in the broad sense. Finally, I will argue that these two approaches represent complementary perspectives that are both needed to make psychology into a mature science.

### The Concept of Person and the Person-Oriented Approach

Zagaria et al. (2020) presents an interesting review of psychological “core-constructs” and how they are defined in textbooks of psychology and in an APA dictionary of Psychology. It is illuminating to see how discordantly various constructs such as “mind” and “behavior” are defined. But it is also significant, and symptomatic, that the concept of person does not appear anywhere in this review. It may well be argued that “person” is a core construct in psychological science – perhaps even *the* core construct.

One early representative of this viewpoint was William Stern (1935) who argued that the concept of person is “psychophysically neutral” and primary in relation to the concepts of “mind” and “body”. A person, as he defined it, is “a living individual, unique whole, which strives toward goals, is self-contained and yet open to the world, experiencing” (Stern 1935, p. 98). Strawson (1959) later came to a similar conclusion by arguing that the concept of a person is a primitive concept (i.e., a concept that cannot be analyzed further), which refers to a particular kind of individuals that have both mental and corporeal characteristics. Among present-day theoretical psychologists, Bergner (2017, p. 83) argues that “the primary focus of the entire field of psychology is persons: their behavior, development, neural functioning, personality, emotion, intelligence, and more”, and Bickhard (2017, p. 5) suggests that psychological science needs to be reformed in terms of the basic assumption that “persons are the loci of psychological phenomena.”

An essential thing about Bergman and Magnusson’s (1997) person-oriented approach is that it is *idiographic* in the sense that it starts from a study of the individual person. Developmental processes are by necessity idiosyncratic, and must be studied at the individual level. This does not, however mean that we should abstain from searching for general regularities. The implication is rather that, although the search for regularities may preferably start at the level of the individual, it should continue by the formulation of hypotheses about such regularities that may be tested in additional individuals.

As pointed out by Lamiell (1998), Hamaker (2012) and others, general laws by definition should apply at the level of the individual, and this is something quite different from the statistical regularities at the aggregate level that are studied in traditional psychological research. Methodologically, this may involve the use of longitudinal data collection, experiential sampling, or ecological momentary assessment, and statistical methods such as time series analysis and cluster analysis.

The person-oriented approach, as defined here, however, is not only idiographic (i.e., focusing on individuals rather than aggregates) but also holistic (focusing on the person as a complex system) and interactional (focusing on the person as an intentional agent in interaction with the environment). At a more general level, the person-oriented approach as developed by Bergman and Magnusson (Bergman and Andersson 2010; Bergman and Magnusson 1997; Magnusson 1999, 2001) is firmly anchored in an explicitly holistic and interactionistic paradigm.

### Holism and Interactionism 

Although Magnusson (1999, 2001) seems to have been the first writer to combine holism and interactionism in one unified theoretical framework, the terms holism and interactionism have a long history in biology, philosophy, psychology and other sciences before that. At the same time, both notions are often misunderstood. In a previous attempt at conceptual clarification (Lundh 2015), *interactionism* was defined as an approach to causality which implies a search for the specific kinds of interactions that take place in a specific area of study, and an attempt to analyze and describe these interactions in detail. *Holism* was defined in terms of assumptions about the relations between a whole and its parts, such as the non-reducibility of the whole to its parts, the non-separability of the parts from the whole, and a system’s capacity for self-organization.

Although interactionism may be applicable to all kinds of disciplines, an application to psychological science implies a focus on clarifying the specific forms of interactions that take place between persons and their environments (including other persons), as distinct from interactions more generally. For example, *one* essential aspect of these specific kinds of interactions is that the person interacts with the environment in the form of an *intentional agent*, with desires, beliefs, intentions and emotions in relation to that environment. At the same time, this aspect must not be overemphasized in relation to other forms of interactions that also take place, which do not involve intentional action.

Applying a holistic perspective to psychological science means to see the individual person as a complex system that should be studied as a whole, not only in terms of its functioning at a given moment, but also over time from a developmental perspective, as a being with a temporal life span. Just as an interactional perspective implies a search for the specific forms of interactions that are involved, a holistic perspective means to search for the specific forms of whole-part relations that are involved.

The question about the possible compatibility between such a person-oriented approach to psychological science and an evolutionary psychology in the broad sense may be divided into the following subquestions: Is a gene-centered evolutionary psychology compatible with a person-oriented approach which focuses on the person as a complex system that needs to be studied (1) as a whole (i.e., holism), (2) as an intentional agent in interaction with its environment (i.e., interactionism), and (3) in terms of his or her individual characteristics and development (i.e., an idiographic focus). This also includes the more overarching question if a gene-centered evolutionary psychology is compatible with a holistic-interactional paradigm in general.

### Genes, Persons and Environments

The relationship between genes and individual persons has been conceptualized in a number of different ways. Dawkins (1976, 2016) sees genes as *replicators* and individual persons as *vehicles*, or “survival machines” for genes. According to this view, the fundamental units of natural selection are the *replicators*, defined as “the basic things that survive or fail to survive, that form lineages of identical copies with occasional random mutations” (Dawkins 2016, p. 253). Basically, a replicator is an entity that passes on its structure largely intact in successive replications, and that changes only slowly due to random mutations. The replicators are what brings continuity to life; they are potentially immortal, as distinct from individual organisms which are deadly creatures with a limited life span.

In his book *The Selfish Gene*, Dawkins (1976) synthesized, extended and popularized the work of previous leading geneticists. The book has been enormously influential, although Dawkins (2016) has expressed some regret for having chosen the expression “the selfish gene” rather than the probably more adequate expression “the immortal gene” as its title. Some of Dawkins’ other conceptualizations have also encountered criticism. One formulation that has been criticized is his notion of individual organisms as *vehicles*, or “survival machines” for genes. Although Dawkins conceptualizes the gene as the fundamental unit of selection, selection is not assumed to act directly upon the gene, but upon “vehicles” in the form of individual organisms. Although this formulation may capture essential aspects of the relation between genes and living individuals, an evolutionary psychology based on a view of individual human beings *merely* as vehicles for genes is hardly compatible with a person-oriented approach to psychological science.

An alternative conceptualization was suggested by Hull (1980), who argued for a distinction between replicators and *interactants*, and defined an interactant as “an entity that interacts as a cohesive whole with its environment in such a way that this interaction causes replication to be differential” (p. 318). In this view, genes are replicators whereas persons (and other living organisms) are interactants. Although Hull’s (1980) conceptualization might seem to be an improvement on Dawkins’ formulation, it may be asked whether genes are not also interacting with their environment. For example, how would a DNA molecule be able to replicate itself without an environment?

The notion of “environment” needs to be clarified here. The environment of genes is not equivalent to the environment of individual organisms, which is by definition external to the organism. In contrast, the environment of genes has to include everything *within* the individual organism, including the rest of the genome, which is relevant to its functioning. Moreover, in a longer time perspective, over the generations, this environment includes the entire gene pool of the species. As Dawkins puts it, the other genes.

“are part of the environment in which each gene survives, in the same way as the weather, predators and prey, supporting vegetation and soil bacteria are part of the environment… In the short term, that means the other members of the genome. In the long term, it means the other genes in the gene pool of the species” (Dawkins 2016, p. x).

If replicators are also a kind of interactants, it follows that individual persons (and other living organisms) cannot be distinguished merely by categorizing them as interactants. Applying an interactionistic paradigm here would mean to search for the specific forms of interaction that characterize the interaction between persons and their environments, *and* the specific forms of interaction that characterize the interaction between genes and their environments, respectively. In other words, what characterizes persons and genes, seen as interactants?

Although persons, as a first approximation, can be defined as a variety of interactants that are intentional agents, it is likely that the nature of persons needs to be specified much more precisely than so, and various attempts have been made in that direction (e.g., Baker 2007; Bergner 2017; Bickhard 2017; Ossorio 2006; Stern 1938). Whatever criteria will turn out to be needed for this purpose, however, there seems to be no a priori reason to doubt that the capacities involved (e.g., for intentional action, self-reflection, communication, empathy, cooperation, etc.) may have been selected during the evolution of human beings as a result of the advantages they conferred. In that sense it seems that a person-oriented approach, with its focus on persons as complex self-organizing intentional systems in interaction with their environment, would be quite compatible with a gene-centered evolutionary psychology.

How, then, are we to characterize genes as interactants? First of all, genes obviously are not intentional agents, although the title of Dawkins’ book *The selfish gene* has sometimes been misinterpreted as involving a misguided anthropomorphic personification that would imply the attribution of intentions to genes. Dawkins, however, is very clear that he is speaking metaphorically.

How then do genes interact with their environment? As described by Dawkins, genes do primarily two things: (1) they replicate themselves, and (2) they act on their environment by constructing proteins, which in turn have effects on other processes in a long causal chain which end up in specific phenotypes. This represents two widely different forms of interaction that produce different kinds of effects: “of the two effects that genes have on the world – manufacturing copies of themselves, and influencing phenotypes – the first is inflexible apart from the rare possibility of mutation; the second may be exceedingly flexible” (Dawkins 1989, p. 21). Arguing against genetic determinism, Dawkins (1989) emphasizes that “the variance we seek to explain will have many causes, which interact in complex ways… Environmental events, both internal and external, may modify the effects of genes” (p. 19).

Morover, the genes are described as critically dependent not only on their environment but also on each other, in a way that seems to be quite compatible with a holistic perspective on genes. If the non-separability of the parts from the whole is a defining characteristic of holistic systems, it would seem that the genome may well be such a system:

The effect of the gene depends on its environment, and this includes other genes. Sometimes a gene has one effect in the presence of a particular other gene, and a completely different effect in the presence of another set of companion genes. The whole set of genes in a body constitutes a kind of genetic climate or background, modifying and influencing the effects of any particular gene. (Dawkins 2016, p. 47)

In view of this “intricate interdependence of genes” (Dawkins 2016, p 30), where “it is almost impossible to disentangle the contribution of one gene from another” (p. 29), Dawkins poses the question why we need to speak of individual genes at all. His answer is that this is because of our sexual reproduction with its crossing-over process during meiosis, which “has the effect of mixing and shuffling genes” (p. 30) so that each new individual who is born is characterized by a uniquely new *combination* of genes. Although each individual’s particular genetic configuration is short-lived and only lasts for one generation, it presupposes the existence of potentially very long-lived genes, the paths of which “constantly cross and recross down the generations” (p. 30). At the same time this points the way to idiographic aspects of genetics, as seen in applications such as genetic genealogy.

### Genetic Genealogy as a Person-Oriented form of Genetics

Genetic genealogy is a discipline that combines DNA testing with traditional genealogical and historical records, for the purpose of inferring how individual persons are related (Bettinger 2019). Although genetic genealogy was first utilized by scientists and historians in the 1990s to identify genealogical connections between public historical figures, it has become something of a popular movement during the last decade since a number of companies have started to offer DNA testing to the general public. By combining various forms of genetic and genealogical triangulation persons may, for example, be able to identify their unknown ancestors (Bettinger 2019; Wayne 2019).

Basically, genetic genealogy rests on the fact that individual persons are not just individuals, but are also *intrinsically related to other persons*, according to genealogical and genetic principles. Genealogically these relations vary in terms of distance (from brothers and sisters to second cousins, third cousins, etc.), and genetically they differ in their degree of overlapping DNA sequences. Although each individual person is unique, persons may be more or less similar to each other, genetically and physiologically, as well as psychologically.

From a genetic point of view, each new person represents a re-combination of DNA from its parents. More specifically, each individual person is characterized idiographically by quite specific DNA sequences on 23 pairs of chromosomes: one pair of sex chromosomes (two X chromosomes in females, and one X chromosome and one Y chromosome in males) and 22 pairs of chromosomes that contain our autosomal DNA. Each person inherits 22 such chromosomes from the mother and 22 from the father, and this autosomal DNA combines into a unique new DNA configuration in each new individual that is born. Although autosomal DNA changes through recombination in each new generation, these changes are not completely haphazard, but tend to leave relatively large DNA segments intact from one generation to the next one. This is what makes it possible to use autosomal DNA in genetic genealogy as a way to find common ancestors and to trace one’s unknown lineages back in time.

Genetic genealogy may be seen as a person-oriented form of genetics, and exemplifies how a science of persons in Stern’s (1938) sense is a much wider discipline than psychological science. In other words, genetic genealogy may be seen as a genetics of the person, which focuses on the unique individual person at the level of genetics.

Genetic genealogy may also be used as a model for thinking about the relation between genes and persons in psychological science. Zagaria et al. (2020) sees evolutionary psychology as a theoretical framework that may serve as an integrative understanding of all kinds of inborn tendencies in human beings. We may refer to these inborn tendencies as “traits”, or “capacities”, or “talents”, or “potentials” or something else, but for simplicity’s sake I will here refer to them here as *potentials*. Suppose that there is a gene for each such potential. What happens during the recombination of autosomal DNA that occurs with the development of each new individual person would then be a re-combination of these psychological potentials into *a completely new psychological profile of potentials that is unique for this particular person*.

Although an evolutionary psychology may help to develop a general understanding of the nature of these various potentials, the understanding of the individually unique profiles of psychological potentials lies clearly beyond the scope of any gene-centered evolutionary psychology, and requires the use of a person-oriented approach. It requires a shift from a “gene eye’s view” (Dawkins 2016) to a person perspective.

## Conclusion

To summarize, the present discussion suggests that a gene-centered evolutionary psychology, where genes are conceptualized in accordance with Dawkins (2016) gene-centered perspective, is quite compatible with the holism, interactionism and idiographic perspective that characterizes a person-oriented approach to psychological science. This indicates that it would be possible to synthesize these two approaches.

Moreover, it may be argued that without such a synthesis an evolutionary psychology will be only “half a psychology”. At best, it will be able to produce a *general* understanding of the nature of the various genetically based psychological potentials that inhere in the human gene pool. But by its very nature it is incapable of understanding the profiles of psychological potentials that are *unique* for each specific individual person, and that develop over time in interaction with the environment. The latter requires a person-oriented approach which focuses on these profiles and their development. Because each such profile is unique and only exists during one specific individual’s life-time it is rather uninteresting from a purely gene-centered perspective, which prefers a focus on “immortal genes” (Dawkins 2016). From a person-oriented perspective, on the other hand, it is precisely this unique profile and its development during one life-time that is of primary interest.

It is possible that person and gene should best be viewed as two opposite poles that represent complementary perspectives on human beings, which are both essential to the development of a mature psychological science.

## Data Availability

Not applicable.
